# Supplementary material for: Supporting cells remove and replace sensory receptor hair cells in a balance organ of adult mice
Source: eLife. 2017 Mar 6;6:e18128. doi: 10.7554/eLife.18128 (PMC5338920; doi:10.7554/eLife.18128)
Supplement: Figure 3—source data 1. — Mean (one standard deviation, SD) and 95% confidence interval (CI) of number of F-actin (phalloidin)-labeled phagosomes per utricle. n, number of mice. DOI: http://dx.doi.org/10.7554/eLife.18128.009 [file elife-18128-fig3-data1.docx]

|  | **CBA/CaJ** | | **C57Bl/6J** | | **Swiss Webster** | |
| --- | --- | --- | --- | --- | --- | --- |
| **Age (weeks)** | **n** | **# Phagosomes**  **Mean** (SD)  [95% CI] | **n** | **# Phagosomes**  **Mean** (SD)  [95% CI] | **n** | **# Phagosomes**  **Mean** (SD)  [95% CI] |
| **3** | 7 | **10.4** (1.8)  [9.1 – 11.8] | 5 | **15.2** (3.0)  [12.6 – 17.8] | 3 | **44.3** (19.5)  [22.3 – 66.4] |
| **5-10** | 6 | **9.8** (1.5)  [8.7 – 11.0] | 3 | **10.0** (1.7)  [8.0 – 12.0] | 8 | **48.9** (14.8)  [38.6 – 59.1] |
| **43-46** | 6 | **10.5** (2.4)  [8.6 – 12.4] | 5 | **15.2** (3.4)  [12.2 – 18.2] | 4 | **67.5** (6.5)  [61.2 – 73.8] |

**Figure 3-source data.** **Quantification of** **phagosomes in the normal utricle of three mouse strains at three ages.** Mean (1 standard deviation, SD) and 95% confidence interval (CI) of number of F-actin (phalloidin)-labeled phagosomes per utricle. n, number of mice.
