# Supplementary material for: Supporting cells remove and replace sensory receptor hair cells in a balance organ of adult mice
Source: eLife. 2017 Mar 6;6:e18128. doi: 10.7554/eLife.18128 (PMC5338920; doi:10.7554/eLife.18128)
Supplement: Figure 4—source data 1. — Mean percentage {(one standard deviation, SD); [95% confidence interval, CI]} of tdTomato-labeled SCs in Plp1-CreERT2:ROSA26tdTomato mice given tamoxifen at 6 weeks (wks) of age and sacrificed 1 or 15 weeks post tamoxifen (7 or 21 weeks of age, respectively). SCs were sampled in two striolar regions and 6 extrastriolar regions in each utricle (see Materials and methods). n = 3 mice at both timepoints. DOI: http://dx.doi.org/10.7554/eLife.18128.011 [file elife-18128-fig4-data1.docx]

| **Utricular Region** | **7 wks (1 wk post Tam)**  **Mean** (SD)  [95% CI] | **21 wks (15 wks post Tam)**  **Mean** (SD)  [95% CI] |
| --- | --- | --- |
| **Striolar** | **68.4%** (1.8%)  [63.9% – 72.9%] | **64.8%** (5.2%)  [52.0% - 77.7%] |
| **Extrastriolar** | **91.7%** (6.1%)  [76.4% - 106.9%] | **85.9%** (3.2%)  [77.9% - 94.0%] |

**Figure 4-source data. Quantification of the percentage of SCs labeled with tdTomato in *Plp1-CreER^T2^:ROSA26^tdTomato^* utricles.** Mean percentage {(1 standard deviation, SD); [95% confidence interval, CI]} of tdTomato-labeled SCs in *Plp1-CreER^T2^:ROSA26^tdTomato^* mice given tamoxifen at 6 weeks (wks) of age and sacrificed 1 or 15 weeks post tamoxifen (7 or 21 weeks of age, respectively). SCs were sampled in two striolar regions and six extrastriolar regions in each utricle (see Materials and Methods). n=3 mice at both timepoints.
