# Supplementary material for: Supporting cells remove and replace sensory receptor hair cells in a balance organ of adult mice
Source: eLife. 2017 Mar 6;6:e18128. doi: 10.7554/eLife.18128 (PMC5338920; doi:10.7554/eLife.18128)
Supplement: Figure 5—source data 1. — (A) Mean (one standard deviation, SD) and 95% confidence interval (CI) of number of PCDH15-CD2-labeled stereocilia bundles per utricle in two strains of mice. (B) Mean (1 SD) and 95% CI of number of ATOH1-GFP-positive cells per utricle. HCs were identified as myosin VIIa-positive cells with nuclei in the apical two-thirds of the epithelium. SCs were identified as myosin VIIa-negative cells whose bodies extend across the entire macular depth, whose nuclei are smaller than HC nuclei, and are positioned near the basal lamina. Unknown cells did not meet criteria for HCs or SCs. n, number of mice. DOI: http://dx.doi.org/10.7554/eLife.18128.013 [file elife-18128-fig5-data1.docx]

**A.**

| **Mouse Strain** | **n** | **PCDH15-CD2-labeled bundles**  **Mean** (SD)  [95% CI] |
| --- | --- | --- |
| **CBA/CaJ** | 5 | **17.2** (4.0)  [12.3 – 22.1] |
| **Swiss Webster** | 4 | **24.0** (8.8)  [9.9 – 38.1] |

**B.**

| **Cell Type** | **n** | **ATOH1-GFP-positive cells**  **Mean** (SD)  [95% CI] |
| --- | --- | --- |
| **HCs** | 3 | **82.3** (25.7)  [53.2 – 111.5] |
| **SCs** | 3 | **8.3** (2.9)  [5.1 – 11.6] |
| **Unknown** | 3 | **8.0** (2.6)  [5.0 – 11.0] |

**Figure 5-source data.** **Quantification of immature HC markers in the normal adult mouse utricle.** **A:** Mean (1 standard deviation, SD) and 95% confidence interval (CI) of number of PCDH15-CD2-labeled stereocilia bundles per utricle in two strains of mice. **B:** Mean (1 SD) and 95% CI of number of ATOH1-GFP-positive cells per utricle. HCs were identified as myosin VIIa-positive cells with nuclei in the apical two-thirds of the epithelium. SCs were identified as myosin VIIa-negative cells whose bodies extend across the entire macular depth, whose nuclei are smaller than HC nuclei, and are positioned near the basal lamina. Unknown cells did not meet criteria for HCs or SCs. n, number of mice.
