# Supplementary material for: Supporting cells remove and replace sensory receptor hair cells in a balance organ of adult mice
Source: eLife. 2017 Mar 6;6:e18128. doi: 10.7554/eLife.18128 (PMC5338920; doi:10.7554/eLife.18128)
Supplement: Figure 6—source data 1. — Mean (one standard deviation, SD) and 95% confidence interval (CI) of the number of tdTomato-labeled HCs per utricle categorized by type in Plp1-CreERT2:ROSA26tdTomato mice given tamoxifen at 6 weeks (wks) of age (right) or in age-matched littermate controls that did not receive tamoxifen (left). Un., unknown. n, number of mice. DOI: http://dx.doi.org/10.7554/eLife.18128.015 [file elife-18128-fig6-data1.docx]

| **No Tamoxifen** | | | **Tamoxifen at 6 wks** | | |
| --- | --- | --- | --- | --- | --- |
| **Age**  **(wks)** | **HC type** | **# labeled HCs**  **Mean** (SD)  [95% CI] | **Time post Tam (wks)** | **HC type** | **# labeled HCs**  **Mean (**SD)  [95% CI] |
| **7**  (n=4) | **I** | **0.8** (1.0)  [-0.2 – 1.7] | **1**  (n=5) | **I** | **4.0** (4.5)  [0.0 – 8.0] |
|  | **II** | **2.8** (3.6)  [-0.8 – 6.3] |  | **II** | **8.5** (4.8)  [4.3 – 12.7] |
|  | **Un.** | **1.0** (0.8)  [0.2 – 1.8] |  | **Un.** | **4.3** (6.0)  [-1.0 – 9.6] |
| **10**  (n=4) | **I** | **4.5** (4.2)  [0.4 – 8.6] | **4**  (n=4) | **I** | **3.0** (1.4)  [1.6 – 4.4] |
|  | **II** | **7.3** (5.7)  [1.6 – 12.9] |  | **II** | **16.3** (3.1)  [13.2 – 19.3] |
|  | **Un.** | **2.5** (2.1)  [0.5 – 4.5] |  | **Un.** | **3.0** (1.8)  [1.2 – 4.8] |
| **16**  (n=6) | **I** | **2.5** (1.9)  [1.0 – 4.0] | **10**  (n=7) | **I** | **7.4** (8.4)  [1.2 – 13.6] |
|  | **II** | **10.7** (4.2)  [7.3 – 14.0] |  | **II** | **27.4** (10.8)  [19.4 – 35.4] |
|  | **Un.** | **2.0** (2.7)  [-0.1 – 4.1] |  | **Un.** | **2.6** (1.7)  [1.3 – 3.8] |
| **21**  (n=4) | **I** | **2.5** (5.0)  [-2.4 – 7.4] | **15**  (n=8) | **I** | **4.1** (2.9)  [2.1 – 6.0] |
|  | **II** | **3.3** (4.0)  [-0.6 – 7.1] |  | **II** | **36.6** (7.7)  [31.3 – 41.9] |
|  | **Un.** | **2.3** (2.2)  [0.1 – 4.4] |  | **Un.** | **7.6** (4.1)  [4.8 – 10.5] |

**Figure 6-source data.** **Quantification of tdTomato-labeled** **HCs in *Plp1-CreER^T2^:ROSA26^tdTomato^* utricles over time.** Mean (1 standard deviation, SD) and 95% confidence interval (CI) of the number of tdTomato-labeled HCs per utricle categorized by type in *Plp1-CreER^T2^:ROSA26^tdTomato^* mice given tamoxifen at 6 weeks (wks) of age (right) or in age-matched littermate controls that did not receive tamoxifen (left). Un., unknown. n, number of mice.
