# Supplementary material for: Supporting cells remove and replace sensory receptor hair cells in a balance organ of adult mice
Source: eLife. 2017 Mar 6;6:e18128. doi: 10.7554/eLife.18128 (PMC5338920; doi:10.7554/eLife.18128)
Supplement: Figure 7—source data 1. — Mean (one standard deviation, SD) and 95% confidence interval (CI) of the number and percentage of tdTomato-labeled HCs per utricle, categorized by type [type I, type II, or unknown (Un.)]. Atoh1-CreERTM:ROSA26tdTomato mice were given tamoxifen at 6 weeks (wks) of age (right) or were age-matched littermate controls that did not receive tamoxifen (left). For the graph in Figure 7G, we present the number of tdTomato-positive type I HCs at 1, 10, 15, and 32 weeks post tamoxifen (shown here), normalized to the total number of tdTomato-positive cells at each timepoint. n, number of mice. DOI: http://dx.doi.org/10.7554/eLife.18128.020 [file elife-18128-fig7-data1.docx]

| **No Tamoxifen** (n=3 for each age group) | | | | **Tamoxifen at 6 wks** (n=4 for each age group) | | | |
| --- | --- | --- | --- | --- | --- | --- | --- |
| **Age**  (wks) | **HC type** | **# labeled HCs**  **Mean** (SD)  [95% CI] | **% labeled HCs**  **Mean** (SD)  [95% CI] | **Time post Tam** (wks) | **HC type** | **# labeled HCs**  **Mean** (SD)  [95% CI] | **% labeled HCs**  **Mean** (SD)  [95% CI] |
| **7** | **I** | **2.3** (2.5)  [-0.5 – 5.2] | **26.2** (13.5)  [7.5 – 44.9] | **1** | **I** | **50.3** (40.0)  [11.1 – 89.4] | **5.9** (3.0)  [2.9 – 8.9] |
|  | **II** | **6.0** (5.3)  [0.0 – 12.0] | **70.2** (18.5)  [44.6 – 95.9] |  | **II** | **688.5** (238.8)  [454.5 – 922.5] | **92.7** (2.8)  [90.0 – 95.4] |
|  | **Un.** | **0.3** (0.6)  [-0.3 – 1.0] | **3.6** (5.1)  [-3.4 – 10.6] |  | **Un.** | **10.3** (5.5)  [4.9 – 15.6] | **1.4** (0.9)  [0.5 – 2.3] |
| **16** | **I** | **1.3** (0.6)  [0.7 – 2.0] | **8.8** (3.6)  [4.7 – 13.0] | **10** | **I** | **74.8** (23.7)  [51.5 – 98.0] | **7.0** (1.4)  [5.6 – 8.4] |
|  | **II** | **12.7** (3.5)  [8.7 – 16.6] | **80.9** (7.9)  [72.0 – 89.8] |  | **II** | **967.8** (123.9) [846.3 – 1089.2] | **91.7** (2.1)  [89.6 – 93.8] |
|  | **Un.** | **2.0** (2.0)  [-0.3 – 4.3] | **10.3** (9.6)  [-0.6 – 21.1] |  | **Un.** | **14.8** (9.4)  [5.5 – 24.0] | **1.4** (0.8)  [0.6 – 2.1] |
| **21** | **I** | **1.0** (1.0)  [-0.1 – 2.1] | **5.4** (4.7)  [0.1 – 10.8] | **15** | **I** | **27.0** (5.0)  [22.1 – 31.9] | **3.7** (0.8)  [2.9 – 4.4] |
|  | **II** | **15.3** (4.5)  [10.2 – 20.4] | **88.5** (7.4)  [80.1 – 96.9] |  | **II** | **711.5** (91.8)  [621.5 – 801.5] | **95.7** (1.1)  [94.6 – 96.8] |
|  | **Un.** | **1.3** (1.5)  [-0.4 – 3.1] | **6.1** (6.0)  [-0.7 – 12.9] |  | **Un.** | **4.8** (4.0)  [0.8 – 8.7] | **0.6** (0.5)  [0.1 – 1.1] |
| **38** | **I** | **5.3** (2.9)  [2.1 – 8.6] | **26.6** (10.9)  [14.2 – 38.9] | **32** | **I** | **92.5** (22.4)  [70.6 – 114.4] | **8.4** (1.8)  [6.7 – 10.2] |
|  | **II** | **14.3** (7.6)  [5.8 – 22.9] | **72.4** (10.5)  [60.5 – 84.2] |  | **II** | **973.5** (114.7)  [861.1 – 1085.9] | **88.9** (1.5)  [87.5 – 90.3] |
|  | **Un.** | **0.3** (0.6)  [-0.3 – 1.0] | **1.1** (1.9)  [-1.0 – 3.2] |  | **Un.** | **28.0** (5.4)  [22.8 – 33.2] | **2.6** (0.7)  [1.9 – 3.3] |

**Figure 7-source data.** **Quantification of tdTomato-labeled** **HCs in *Atoh1-CreER^TM^:ROSA26^tdTomato^* utricles.** Mean (1 standard deviation, SD) and 95% confidence interval (CI) of the number and percentage of tdTomato-labeled HCs per utricle, categorized by type [type I, type II, or unknown (Un.)]. *Atoh1-CreER^TM^:ROSA26^tdTomato^* mice were given tamoxifen at 6 weeks (wks) of age (right) or were age-matched littermate controls that did not receive tamoxifen (left). For the graph in Fig. 7G, we present the number of tdTomato-positive type I HCs at 1, 10, 15, and 32 weeks post tamoxifen (shown here), normalized to the total number of tdTomato-positive cells at each timepoint. n, number of mice.
