# Supplementary material for: Supporting cells remove and replace sensory receptor hair cells in a balance organ of adult mice
Source: eLife. 2017 Mar 6;6:e18128. doi: 10.7554/eLife.18128 (PMC5338920; doi:10.7554/eLife.18128)
Supplement: Figure 8—source data 1. — Mean (one standard deviation, SD) and 95% confidence interval (CI) of the number and percentage of tdTomato-labeled HCs per utricle. Plp1-CreERT2:ROSA26tdTomato:Pou4f3DTR mice (damaged) were given tamoxifen at 9 weeks of age, DT at 10 weeks of age and analyzed at 13 weeks of age. Controls were littermates that did not contain the Pou4f3DTR allele but received both the tamoxifen and DT injections. n, number of mice. DOI: http://dx.doi.org/10.7554/eLife.18128.022 [file elife-18128-fig8-data1.docx]

| **Genotype** | **n** | **# labeled HCs**  **Mean** (SD)  [95% CI] | **% labeled HCs**  **Mean** (SD)  [95% CI] |
| --- | --- | --- | --- |
| Control | 3 | **16.3** (4.5)  [5.1 – 27.5] | **0.4%** (0.1%)  [0.3% – 0.6%] |
| Damaged | 4 | **101.3** (49.7)  [22.2 – 180.3] | **23.9%** (8.2%)  [15.9% – 31.9%] |

**Figure 8-source data.** **Quantification of tdTomato-labeled HCs after HC damage** **in *Plp1-CreER^TM^:ROSA26^tdTomato^:Pou4f3^DTR^* and control utricles.** Mean (1 standard deviation, SD) and 95% confidence interval (CI) of the number and percentage of tdTomato-labeled HCs per utricle. *Plp1-CreER^TM^:ROSA26^tdTomato^:Pou4f3^DTR^*  mice (damaged) were given tamoxifen at 9 weeks of age, DT at 10 weeks of age and analyzed at 13 weeks of age. Controls were littermates that did not contain the *Pou4f3^DTR^* allele but received both the tamoxifen and DT injections. n, number of mice.
