# Supplementary material for: Supporting cells remove and replace sensory receptor hair cells in a balance organ of adult mice
Source: eLife. 2017 Mar 6;6:e18128. doi: 10.7554/eLife.18128 (PMC5338920; doi:10.7554/eLife.18128)
Supplement: Figure 9—source data 1. — Mean (one standard deviation, SD) and 95% confidence interval (CI) of number of F-actin (phalloidin)-labeled phagosomes per utricle. Littermates lacking the Pou4f3DTR allele were used as control and labeled as 0 day post DT. n, number of mice. DOI: http://dx.doi.org/10.7554/eLife.18128.024 [file elife-18128-fig9-data1.docx]

|  | ***Pou4f3^DTR^*** | |
| --- | --- | --- |
| **Time post DT (days)** | **n** | **# Phagosomes**  **Mean** (SD)  [95% CI] |
| 0 | 4 | **9.5** (1.7)  [6.7 – 12.3] |
| 4 | 3 | **15.3** (2.1)  [10.2 – 20.5] |
| 7 | 7 | **15.3** (2.9)  [12.6 – 17.9] |
| 14 | 4 | **12.0** (2.5)  [8.1 – 15.9] |
| 40 | 4 | **7.8** (2.4)  [4.0 – 11.5] |
| 90 | 4 | **7.3** (3.5)  [1.7 – 12.8] |
| 120 | 3 | **8.0** (2.0)  [3.0 – 13.0] |

**Figure 9-source data.** **Quantification of** **phagosomes in *Pou4f3^DTR^* mice after HC damage.** Mean (1 standard deviation, SD) and 95% confidence interval (CI) of number of F-actin (phalloidin)-labeled phagosomes per utricle. Littermates lacking the *Pou4f3^DTR^* allele were used as control and labeled as 0 day post DT. n, number of mice.
